# Anti-Inflammatory Activity-Guided Isolation and In Silico Validation of Turmeric (*Curcuma longa* L.) Phytochemicals

**DOI:** 10.3390/foods14244205

**Published:** 2025-12-07

**Authors:** Zhuldyz Uvaniskanova, Salar Hafez-Ghoran, Muhammad Ikhlas Abdjan, Bel Youssouf G. Mountessou, Fatemeh Taktaz, Fadjar Mulya, Gulnaz A. Seitimova, Muhammad Iqbal Choudhary

**Affiliations:** 1 Faculty of Chemistry and Chemical Technology, Al-Farabi Kazakh National University, Almaty 050040, Kazakhstan; 2 Laboratory for Functional Foods and Human Health, Center for Excellence in Post-Harvest Technologies, North Carolina Research Campus, North Carolina Agricultural and Technical State University, 500 Laureate Way, Kannapolis, NC 28081, USA; 3 H.E.J. Research Institute of Chemistry, International Center for Chemical and Biological Sciences (ICCBS), University of Karachi, Karachi 75270, Pakistan; 4 Department of Chemistry, Faculty of Mathematics and Natural Science, Universitas Negeri Surabaya, Surabaya 60213, Indonesia; 5 Department of Chemistry, Higher Teacher Training College, University of Yaoundé I, Yaoundé P.O. Box 47, Cameroon; 6 Nanotechnology Engineering, Faculty of Advanced Technology and Multidiscipline, Airlangga University, Surabaya 60115, Indonesia; 7 Dr. Panjwani Center for Molecular Medicine and Drug Research (PCMD), International Center for Chemical and Biological Sciences (ICCBS), University of Karachi, Karachi 75270, Pakistan

**Keywords:** *Curcuma longa* L., β-turmerone, bioassay-guided isolation, anti-inflammatory activity, dynamic simulations

## Abstract

Turmeric (*Curcuma longa* L., Zingiberaceae) is a widely consumed spice and functional food valued for its bioactive constituents. Using an activity-guided strategy, this study identified the dichloromethane fraction as the most potent anti-inflammatory fraction, exhibiting markedly stronger inhibition of reactive oxygen species (ROS) production than ibuprofen (IC_50_ ≤  0.4 vs. 11.2 μg/mL). Bioassay-guided purification yielded bisacurone (**1**), didemethoxycurcumin (**2**), and β-turmerone (**3**), with compounds **1** and **2** reported here for the first time in this fraction. Among them, β-turmerone displayed the strongest anti-inflammatory activity (IC_50_ = 4.7 μg/mL), consistent with in silico docking and molecular dynamics analyses, revealing greater binding affinity and complex stability with myeloperoxidase (ΔG_bind_ = −20.90 vs. −18.89 kcal/mol for ibuprofen). Gas chromatography–mass spectrometry (GC-MS) profiling revealed a phytochemical profile dominated by turmerones and curlone, correlating with the observed bioactivity. None of the fractions exhibited acute toxicity in brine shrimp lethality assays, indicating a favorable preliminary safety profile. Our findings demonstrate the value of activity-guided isolation combined with computational validation for identifying turmeric-derived bioactives with promising nutraceutical potential, warranting further in vivo evaluation.

## 1. Introduction

Turmeric (*Curcuma longa* L., Zingiberaceae) originates from Southwest India, and its rhizomes serve as the source of a vibrant yellow spice with broad culinary, medicinal, and industrial applications. It is now extensively cultivated across tropical regions and is used not only as a therapeutic agent but also in the cosmetic industry and as a natural dye [1]. Although approximately 93 to 100 *Curcuma* species have been described, the exact number remains debated; among them, *C. longa* (turmeric) and *C. zedoaria* (zedoary) are the most extensively studied due to their high commercial and pharmacological value [2,3]. Owing to its wide-ranging bioactivities, turmeric has attracted considerable global attention in recent years. Traditionally, *C. longa* rhizomes have been used across China, Japan, Pakistan, Bangladesh, and Southeast Asia as carminative, stomachic, anthelmintic, and laxative agents, and in the treatment of liver disorders [3,4,5]. The biological activity of turmeric has long been attributed chiefly to its curcuminoids (e.g., curcumin, demethoxycurcumin, bisdemethoxycurcumin), which are well known for their antitumor, antioxidant, anti-inflammatory, anti-HIV, anti-Alzheimer’s, hepatoprotective, and cardiovascular-protective properties [5,6,7,8,9]. However, turmeric also contains an extensive spectrum of non-curcuminoid phytochemicals, particularly in its essential oil, that contribute significantly to its pharmacological effects. Major constituents of turmeric oil include sesquiterpenoids such as ar-turmerone, α-turmerone, and β-turmerone, as well as α-zingiberene, ar-curcumene, santalenone, β-sesquiphellandrene, and β-bisabolene [10,11].

Growing evidence demonstrates that these volatile and non-curcuminoid components possess potent anti-inflammatory and anti-neuroinflammatory properties. For example, ar-turmerone suppresses neuroinflammation in Aβ-stimulated BV2 microglial cells by downregulating pro-inflammatory mediators, including cyclooxygenase 2 (COX-2), iNOS, TNF-α, IL-1β, IL-6, and MCP-1, reducing intracellular reactive oxygen species (ROS) levels, and inhibiting NF-κB, JNK, and p38 MAPK signaling pathways; it also protects hippocampal neurons from microglia-mediated cytotoxicity [12,13]. Likewise, curcumin-free turmeric extracts enriched in turmerone, bisacurone, calebin A, and germacrone have exhibited strong anti-inflammatory, anticancer, and antidiabetic activities, confirming that non-curcuminoid fractions contribute substantially to turmeric’s multifaceted health effects [14,15].

Despite these promising findings, research on *Curcuma longa* L. (turmeric) remains predominantly centered on curcumin, and non-curcuminoid compounds have received disproportionately limited attention. To address this gap, the present study focuses on the non-polar fractions of *C. longa* and integrates anti-inflammatory activity-guided fractionation with structural elucidation of isolated phytochemicals using advanced spectroscopic techniques (NMR and MS), GC-MS profiling of bioactive fractions, and in silico computational validation.

## 2. Materials and Methods

### 2.1. General Experiment Procedures

Nuclear magnetic resonance (NMR) spectra were acquired on a Bruker AV-500 spectrometer (Bruker Inc., Fällanden, Switzerland). Mass spectrometric analyses, including low-resolution fast atom bombardment (LR-FAB-MS) and low-resolution electron ionization (LR-EI-MS), were performed using a JEOL MS Route JMS 600H instrument (JEOL Ltd., Akishima, Japan). Column chromatography was conducted on normal-phase silica gel (Merck, 70–230 mesh), with further purification achieved using C18 silica (Wakogel, 38–63 mesh), Sephadex LH-20 (GE Healthcare, Chicago, IL, USA), and both normal-phase and reverse-phase high-performance liquid chromatography (HPLC) systems. Sample purity was assessed on precoated thin-layer chromatography (TLC) plates under normal and reverse-phase conditions. TLC spots were visualized under UV light at 254 and 366 nm and using ceric sulphate and vanillin/sulfuric acid spray reagents. Chemical profiling of bioactive fractions was carried out by gas chromatography–mass spectrometry (GC-MS) using an Agilent 7890N GC system coupled to an Agilent Triple Quad 7000A mass spectrometer operating in EI mode at 70 eV (Agilent, Palo Alto, CA, USA).

### 2.2. Plant Materials

*Curcuma longa* rhizomes were collected from Pakistan and taxonomically authenticated by Dr. Muneeba Khan at the Herbarium—Center for Plant Conservation, University of Karachi. A voucher specimen (G.H. No 99723) was deposited in the same Center for further reference and conservation.

### 2.3. Extraction and Isolation

Fresh rhizomes of *Curcuma longa* L. (15 kg) were cleaned, washed with deionised water, sliced, and air-dried under sunlight for one week. The dried material was then cut into small pieces and ground using an electronic mill under standardized conditions to ensure uniform particle size suitable for solvent extraction; although the exact particle diameter was not measured, identical processing conditions were applied to maintain batch-to-batch consistency. The resulting powder (3.5 kg) was exhaustively macerated with 70% ethanol (3×) at room temperature. The combined extracts were concentrated under reduced pressure using a rotary evaporator to yield a dark brown crude residue (583 g). This extract was suspended in distilled water and successively partitioned with *n*-hexane (68 g), dichloromethane (DCM, 52 g), ethyl acetate (EtOAc, 70 g), *n*-butanol (26 g), and finally water, affording five fractions; the masses obtained for each are indicated in parentheses.

**Figure 1:**
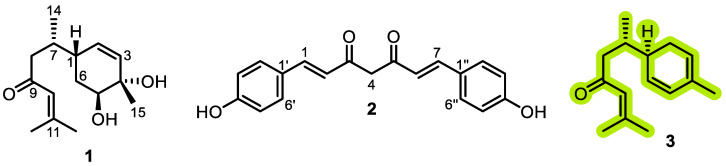
Chemical structures of compounds isolated from the dichloromethane fraction of *Curcuma longa* L.

Bioassay-guided fractionation based on anti-inflammatory activity identified the dichloromethane fraction as the most active. Approximately 25 g of this fraction was adsorbed onto silica gel (G60, 0.2–0.5 mm) and subjected to open column chromatography (CC) using DCM as the initial eluent, followed by a stepwise DCM-methanol gradient (up to 10% methanol). This procedure yielded 198 individual fractions, which were consolidated into six major groups (A–F) based on their TLC characteristics (R_f_ values, UV_254_/UV_366_ fluorescence, and vanillin/sulfuric acid reagent). Fraction D (~850 mg) was selected for further purification due to its chemically rich profile. It was fractionated on a silica gel column (40 cm × 3 cm, packed with 100 g silica gel G60) using a gradient of *n*-hexane-acetone (9:1 → 1:1, *v*/*v*). This separation afforded compound **1** (2.8 mg) in pure form from one set of eluates. Another set of fractions eluted with *n*-hexane–acetone (7:3, *v*/*v*) was combined to yield sub-fraction D_1_ (150 mg). Sub-fraction D_1_ was subjected to final purification using a recycling preparative HPLC system (Japan Analytical Industry Co., Ltd., LC-908) equipped with a JAIGEL-ODS-M-80 column (250 mm × 20 mm, 4 μm, 80 Å). Isocratic elution with methanol–water (80:20, *v*/*v*) at a flow rate of 4.0 mL/min yielded compounds **2** (9.1 mg) and **3** (12.7 mg) (Figure 1).

### 2.4. Spectroscopy Data

#### 2.4.1. Bisacurone (**1**)

White solid, C_15_H_24_O_3_; ^1^H NMR (600 MHz, CD_3_OD) *δ_H_* 6.19 (1H, *s*, H-10), 5.62 (1H, *dd*, 2.3; 10.2 Hz, H-2), 5.54 (1H, *dd*, 1.3; 10.2 Hz, H-3), 3.68 (1H, *dd*, 1.9; 6.4 Hz, H-5), 2.27 (1H, *m*, H-1), 2.53 and 2.23 (2H, *dd*, 4.9; 15.3 Hz, H-8), 2.12 (1H, *m*, H-7), 2.11 (3H, *s*, H-12), 1.90 (3H, *s*, H-13), 1.82 and 1.65 (2H, *m*, H-6), 1.23 (3H, *s*, H-15), 0.89 (3H, d, 6.9 Hz, H-14); ^13^C NMR (150 MHz, CD_3_OD) *δ_C_* 203.5 (C-9), 157.2 (C-11), 133.5 (C-3), 132.5 (C-2), 125.0 (C-10), 74.0 (C-5), 70.8 (C-4), 49.7 (C-8), 37.7 (C-1), 34.6 (C-7), 29.3 (C-6), 20.9 (C-12), 27.7 (C-13), 24.8 (C-15), 17.2 (C-14); (+)-FAB-MS *m*/*z* 253.2 [M+H]^+^ (Supplementary Figures S1–S3) [16].

#### 2.4.2. Didemethoxycurcumin (**2**)

Yellow solid, C_19_H_16_O_4_; ^1^H NMR (500 MHz, CD_3_OD) *δ*_H_ 7.57 (2H, d, 16.0 Hz, H-1/H-7), 6.59 (2H, *d*, 16.0 Hz, H-2/H-6), 7.49 (4H, *d*, 8.5 Hz, H-2′/H-6′ and H-2′′/H-6′′), 4.57 (2H, *s*, H-4), 6.81 (4H, *d*, 8.5 Hz, H-3′/H-3′ and H-3′′/H-3′′); ^13^C NMR (125 MHz, CD_3_OD) *δ*_C_ 199.2 (C-3/C-5), 159.2 (C-4′′/C-4′′), 132.8 (C-1/C-7), 131.4 (C-2/C-6), 130.2 (C-2′/C-6′/C-2′′/C-6′′), 129.3 (C-1′/C-1′′), 116.7 (C-3′/C-5′/C-3′′/C-5′′), 53.2 (C-4); LR-EI-MS *m*/*z* (rel. int., %): 308 (10), 290 (14), 120 (100), 91 (64), 44 (58) (Supplementary Figures S4–S6) [17].

#### 2.4.3. β-Turmerone (**3**)

White solid, C_15_H_22_O; ^1^H NMR (500 MHz, CD_3_OD) *δ*_H_ 6.26 (1H, *dd*, 2.0; 10.0 Hz, H-3), 6.18 (1H, *s*, H-10), 5.74 (1H, *d*, 10.2 Hz, H-6), 5.60 (1H, *dd*, 1.9; 10.2 Hz, H-5), 2.93 and 1.98 (2H, *dd*, 2.3; 10.2 Hz, H-8), 2.19 and 1.93 (2H, *m*, H-2), 2.12 (3H, *s*, H-15), 2.11 (3H, *s*, H-12), 1.92 (1H, *m*, H-1), 1.90 (3H, *s*, H-13), 1.70 (1H, *m*, H-7), 0.89 (3H, *d*, 6.9 Hz, H-14); ^13^C NMR (125 MHz, CD_3_OD) *δ*C 203.3 (C-9), 157.2 (C-11), 134.1 (C-6), 132.0 (C-4), 125.0 (C-10), 124.1 (C-5), 121.1 (C-3), 49.8 (C-8), 38.1 (C-1), 34.3 (C-7), 29.9 (C-2), 27.7 (C-13), 21.5 (C-15), 20.9 (C-12), 19.9 (C-14); (+)-FAB-MS *m*/*z* 219.2 [M+H]^+^ (Supplementary Figures S7–S9) [18].

### 2.5. GC-MS Analysis of the Active Fractions

The composition of the essential oils was analyzed by GC-MS using an Agilent 7890N gas chromatograph coupled to an Agilent Triple Quad 7000A mass spectrometer operated in EI mode at 70 eV (scan range *m*/*z* 40–600), with the ion source temperature set at 250 °C. Data acquisition was performed using Agilent ChemStation software. Chromatographic separation achieved on an Agilent DB-35-MS capillary column (30 m × 0.32 mm × 0.25 μm). The injector operated in split/splitless mode at 200 °C, and detection was performed with a flame ionization detector (FID) maintained at 230 °C. The oven temperature program was as follows: initial temperature 50 °C (held 5 min), ramped at 3 °C/min to 200 °C (held 20 min), then increased at 10 °C/min to 300 °C (held 15 min). Helium was used as the carrier gas at a constant flow rate of 1.5 mL/min. Identification of constituents was based on comparison of mass spectra with the NIST (1998) library and evaluation of retention indices (RI) relative to literature values. Quantification was performed by calculating the relative percentage of each compound from its peak area in the total ion chromatogram. Retention indices were determined by interpolation using a homologous series of *n*-alkanes analyzed under identical chromatographic conditions [19].

### 2.6. Anti-Inflammatory Assay

A luminol-enhanced chemiluminescence assay was conducted as described by Mirahmad et al. (2024) [20]. Briefly, 25 µL of diluted whole blood in HBSS++ (Sigma-Aldrich, St. Louis, MO, USA) was incubated with 25 µL of the test compounds (1, 10, and 100 µg/mL) in triplicate. Control wells contained HBSS++ and cells only. The assay was carried out in white half-area 96-well plates (Costar, New York, NY, USA) at 37 °C for 15 min within the thermostatted chamber of a luminometer (Labsystems, Helsinki, Finland). Subsequently, 25 µL of serum-opsonized zymosan (SOZ; Fluka, Buchs, Switzerland) and 25 µL of luminol (Research Organics, Cleveland, OH, USA) were added to each well, except for blank wells containing HBSS++ alone. ROS production was recorded as relative light units (RLU). Ibuprofen was used as the standard reference compound.

### 2.7. Brine Shrimp Lethality Test (BSLT)

Cytotoxicity assays were conducted on the fractions of *C*. *longa* using the *Artemia salina* (brine shrimp) lethality bioassay following established procedures [20]. (a) Test Materials: Brine shrimp eggs (*A. salina*), sea salt solution (38 g/L, pH 7.4), hatching tray, illumination lamp, micropipettes, organic solvents (methanol, ethanol, acetone), and distilled water were used. (b) Storage and Hatching: Eggs were stored at 4 °C and hatched by dispersing 50 mg into a hatching tray containing brine solution. The tray was incubated at 37 °C under continuous illumination for 48 h to obtain active nauplii. (c) Sample Preparation and Bioassay: Test samples (20 mg) were dissolved in 2 mL solvent, and aliquots of 5, 50, and 500 µL were transferred to vials to yield final concentrations of 10, 100, and 1000 µg/mL. Solvent was allowed to evaporate overnight before adding nauplii (10 per vial). The volume was adjusted to 5 mL with seawater, and the vials were incubated at 25−27 °C for 24 h under illumination. Negative controls contained solvent only, while a standard cytotoxic drug served as the positive control. (d) Data Analysis: Mortality was recorded after 24 h, and LD_50_ values were calculated using Finney’s probit analysis program.

Since this assay involves invertebrate larvae, it is not classified as an animal experiment under current international ethical guidelines (e.g., EU Directive 2010/63/EU, OECD principles); therefore, formal animal ethics approval was not required. The assay was nonetheless conducted following established laboratory safety and ethical standards.

### 2.8. Computational Detail

#### 2.8.1. Data Preparation

The crystal structure of human myeloperoxidase (MPO, PDB ID: 1DNU) was selected as the targeted protein [21]. The structure contains a HEME cofactor located in the active site. Amino acid residues and the HEME moiety were extracted from the crystal structure and prepared as targets for small-molecule inhibition. Protonation of amino acid residues was performed at pH 7.0 using the H++ web server (http://newbiophysics.cs.vt.edu/H++/, assessed on 1 January 2022). Small molecules, including Ibuprofen (Ibu) as a reference and the selected isolated compound, were constructed and geometry-optimized using density functional theory (DFT) at the B3LYP/6-311++G(d,p) level with the Gaussian 16 software package [22]. Missing parameters for bonded, nonbonded, and restrained charges were generated using Antechamber with the Amber FF14SB force field.

#### 2.8.2. Molecular Docking

Molecular docking of the target protein was performed using the DOCK6 package following the standard protocol [23]. The docking workflow utilized multiple tools within DOCK6, including grid-box construction via the *sphere_selector* tool, based on the coordinates of the cluster spheres. Key parameters for grid generation include a *grid spacing* of 0.3 Å, a *grid center* at (x: 16.87, y: −20.97, z: 2.25), and *grid dimensions* of (x: 27.21, y: 26.05, x: 31.43). Ligand–receptor interactions were evaluated using the *Crippen–Kuhl geometric matching* algorithm to calculate the grid score (GS) [24], defined as the sum of van der Waals (E_vdW_) and electrostatic (E_ele_) energies under gas-phase conditions [25]. The resulting docking poses provided initial ligand–protein complex conformations for further molecular dynamics (MD) simulations.

#### 2.8.3. Molecular Dynamics (MD) Simulation

MD simulations were performed using the Amber25 package. The simulation systems (Apo and ligand-bound complexes) were prepared with the *tleap* tool [26] to generate neutral, solvated systems. Solvation employed the TIP3PBOX water model with a 12 Å buffer and chloride ions (Cl^−^) for neutralization [27]. Energy minimization was conducted in three gradual stages to relieve unfavorable atomic contacts and steric clashes: (i) water and ions, (ii) Apo/complex, and (iii) the entire system. Each stage combined steepest descent (maxcyc: 1500 steps) and conjugate gradient (ncyc: 500 steps) algorithms. The system was then gradually heated from 10 K to 310 K (−263 °C to 37 °C; time step: 1500 ps) using the canonical ensemble (NVT). Reporting temperature in Kelvin facilitates computational thermodynamic calculations, while the corresponding Celsius values are provided for clarity and comparison with experimental conditions. Subsequently, the system was equilibrated four times at 310 K (~37 °C; time step: 1300 ps), with harmonic restraints of 30, 20, 10, and 5 kcal/mol/Å^2^. After heating and equilibration, production MD simulations were performed for 100 ns under periodic boundary conditions at 1 atm and 310 K (~37 °C) using the isothermal–isobaric ensemble (NPT). The generated trajectories were analyzed to calculate root-mean-square deviation (RMSD), root-mean-square fluctuation (RMSF), radius of gyration (RoG), and binding free energy (∆G_bind_). The ∆G_bind_ was estimated using the last 10 ns (90–100 ns) of trajectories via the Molecular Mechanics–Poisson–Boltzmann Surface Area (MM-PBSA) approach [28].

### 2.9. Data Analysis

The biological assays were performed in triplicate, and the results are expressed as mean ± standard deviation (SD).

## 3. Results and Discussion

### 3.1. Isolation of Bioactive Compounds

Through repeated chromatographic separations combined with bioassay-guided fractionation based on anti-inflammatory activity, three compounds were isolated from the active DCM fraction: bisacurone (**1**), didemethoxycurcumin (**2**), and β-turmerone (**3**) (Figure 1). The chemical structures of the isolated compounds were elucidated using mass spectrometry and NMR spectroscopy (Supplementary Figures S1–S9), and were confirmed by comparison with previously reported spectral data [16,17,18].

### 3.2. Bioactivities of Curcuma longa L. Fractions and Isolated Compounds

#### 3.2.1. Anti-Inflammatory Activity

Fractions and isolated compounds from *Curcuma longa* L. rhizomes were evaluated for their ability to inhibit reactive oxygen species (ROS) production. Among the fractions, the non-polar DCM fraction showed the highest potency; its IC_50_ could not be precisely determined due to assay resolution limits, but the assay-reported threshold value obtained (≥0.4 µg/mL) indicates high activity and is conservatively reported here as IC_50_ ≤ 0.4 µg/mL. The *n*-hexane fraction also showed notable activity (IC_50_ of 5.4 µg/mL). Both fractions were more potent than the reference non-steroidal anti-inflammatory drug ibuprofen (IC_50_ of 11.2 µg/mL). Although the EtOAc and *n*-butanol fractions also exhibited measurable activity (IC_50_ of 1.3 and 8.7 µg/mL, respectively), they were not pursued further because the study specifically focused on non-polar fractions. The aqueous fraction was inactive. Of the pure compounds, compound **1** was not tested due to limited availability, whereas compound **3** displayed potent activity (IC_50_ = 4.7 ± 0.6 µg/mL), exceeding that of ibuprofen (Table 1).

These results aligned with previous findings demonstrating that the organic extracts of *C. longa* possess strong anti-inflammatory and antioxidant potential. Lantz et al. reported that turmeric extracts effectively suppressed pro-inflammatory mediators such as PGE_2_ and TNF-α with IC_50_ values of 0.92 and 15.2 µg/mL, respectively, highlighting the role of solvent polarity in the extraction of active components [29]. The pronounced activity of the DCM fraction in the present study likely reflects the synergistic effects of multiple bioactive metabolites, including curcuminoids, turmerones, and phenolics. This hypothesis is supported by Edwards et al., who observed that isolated bisdemethoxycurcumin (BDMC) exhibited lower NF-κB inhibitory activity (IC_50_ = 8.3 µM) than the unfractionated extract, highlighting the importance of matrix interactions [30]. Similarly, Ravindran et al. reported that bisdemethylcurcumin (BDC) more effectively inhibited TNF-α-induced NF-κB activation than curcumin itself, following the potency order BDC = hispolon > hispolon methyl ether > curcumin, suggesting that specific hydroxyl/methoxy substitutions modulate bioactivity [31]. Beyond curcuminoids, several studies have highlighted the contribution of non-curcuminoid components. Bagad et al. revealed that the oil-free aqueous extract of *C. longa* (COFAE) significantly reduced xylene-induced ear edema (36.7–51.2%) and cotton pellet-induced granuloma formation (27–48%) at oral doses of 100–400 mg/kg (*p* ≤ 0.05), with efficacy comparable to both curcuminoids and turmerones [32]. Similarly, the polysaccharide-rich fraction (F1) produced 48.5% inhibition of carrageenan-induced paw edema (45 mg/kg), 42.3% suppression in xylene-induced ear edema (63 mg/kg), and 39.7% reduction in granuloma weight, confirming anti-inflammatory potential beyond phenolic constituents [33].

#### 3.2.2. Cytotoxicity Activity (Brine Shrimp Lethality Assay)

The cytotoxicity of *C. longa* L. fractions was evaluated using the brine shrimp lethality assay (BSLA). None of the tested fractions (*n*-hexane, DCM, EtOAc, *n*-butanol, and aqueous) induced mortality in *Artemia salina* nauplii at concentrations of 10, 100, and 1000 µg/mL (Supplementary Table S1), indicating a favorable safety profile for potential pharmacological and nutraceutical applications. These findings are consistent with previous reports demonstrating the low toxicity of *C. longa* extracts. However, contrasting outcomes were observed in other *Curcuma* species. For instance, phytosomal formulations of standardized *C. longa* extracts showed measurable cytotoxicity in BSLA, likely reflecting possible alterations in bioactive compound bioavailability and delivery [34]. In contrast, ethanolic extracts of *C. aeruginosa* (black turmeric) from diverse Indonesian regions consistently exhibited cytotoxic effects, regardless of curcuminoid levels (0.01–1.95%) [35], suggesting that species-specific metabolites beyond curcuminoids may contribute to the observed toxicity.

### 3.3. Phytochemical Identification Through GC-MS Analysis

Following the DCM fraction, the *n*-hexane fraction exhibited the second-highest anti-inflammatory activity and was further analyzed by GC-MS. Chromatographic profiling identified 30 phytochemicals in the *n*-hexane fraction and 34 in the DCM fraction (Figures S10 and S11, Tables S2 and S3). The dominant constituents in both fractions were aromatic turmerone (30.07% in *n*-hexane; 46.74% in dichloromethane), ar-turmerone (26.57%; 16.02%), and curlone (16.47%; 17.03%). The *n*-hexane fraction was particularly enriched in sesquiterpenes, including zingiberene (3.5%), β-sesquiphellandrene (3.4%), β-caryophyllene (1.26%), and α-santalol (1.2%), whereas the DCM fraction contained minor quantities of dihydrocurcumene (2.07%).

These results closely align with previously reported GC-MS profiles of *C. longa* rhizome extracts. Kirmani et al. identified ar-turmerone (34.63%) as the predominant compound in *n*-hexane extract, accompanied by curlone and β-sesquiphellandrene in smaller proportions [36]. Likewise, Lee et al. compared *n*-hexane extracts from multiple *Curcuma* cultivars using GC-TOF-MS and found α-turmerone, ar-turmerone, curlone, zingiberene (4.6–13.9%), and β-sesquiphellandrene (4.6–10.0%) as major volatiles, highlighting chemotypic consistency across regions [37]. A comprehensive volatile profiling study by Poudel et al. also reported comparable concentrations of turmerone derivatives in *C. longa* essential oils, with ar-turmerone (25.5%), α-turmerone (24.4%), and β-turmerone (14.0%) as principal constituents, together with zingiberene (4.8%), β-sesquiphellandrene (5.1%), and β-caryophyllene (2.9%) [15]. Micheal et al. similarly identified 19 volatiles in *n*-hexane extracts dominated by turmerone-type sesquiterpenes, including curlone [38].

Comparative analysis revealed that solvent polarity markedly influenced the phytochemical composition of *C*. *longa* extracts. The *n*-hexane fraction was enriched in volatile sesquiterpenes, whereas the DCM fraction contained higher levels of oxygenated turmerones. These results highlight the importance of solvent selection in optimizing extraction efficiency and determining the relative abundance of bioactive compounds in *C*. *longa*.

### 3.4. Computational Studies

#### 3.4.1. Docked Conformation

**Figure 2:**
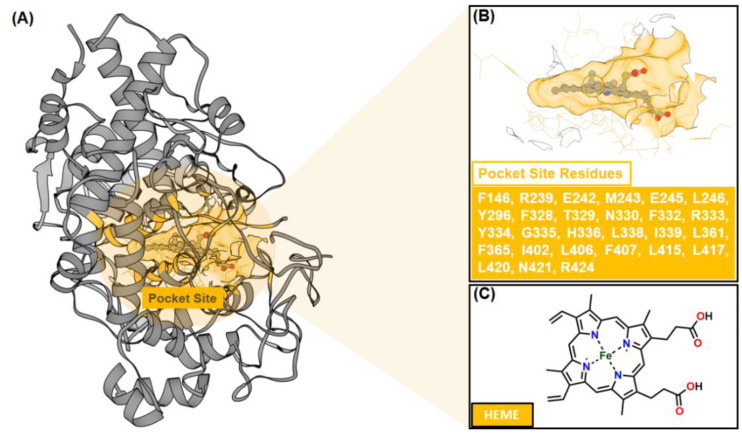
The crystal structure of myeloperoxidase (MPO) as a targeted protein (PDB ID: 1NDU): (**A**) The co-crystal structure has a co-factor (HEME) that binds to the pocket site. (**B**) The pocket site has several amino acids (27 residues). The chemical structure of (**C**) HEME.

Molecular docking was employed to predict the binding conformation and interactions of inhibitors (ligands) within the active site of the target protein (receptor) [39]. This computational approach provides molecular-level insights into inhibitory mechanisms and complements experimental findings. Based on in vitro anti-inflammatory assays (Table 1), compound **3** (C3) exhibited the strongest inhibitory activity among the tested compounds, surpassing ibuprofen. Accordingly, molecular docking was performed to elucidate its potential interactions with myeloperoxidase (MPO), a key enzyme involved in the formation of ROS, such as hypochlorous acid, which contributes to inflammatory responses [21,40,41]. MPO was selected as the docking target because inhibition of its catalytic activity can attenuate ROS production and downstream tissue damage. Moreover, the availability of a high-resolution MPO–heme crystal structure provides a well-defined active site, facilitating rational docking and interpretation of ligand–protein interactions (Figure 2).

**Figure 3:**
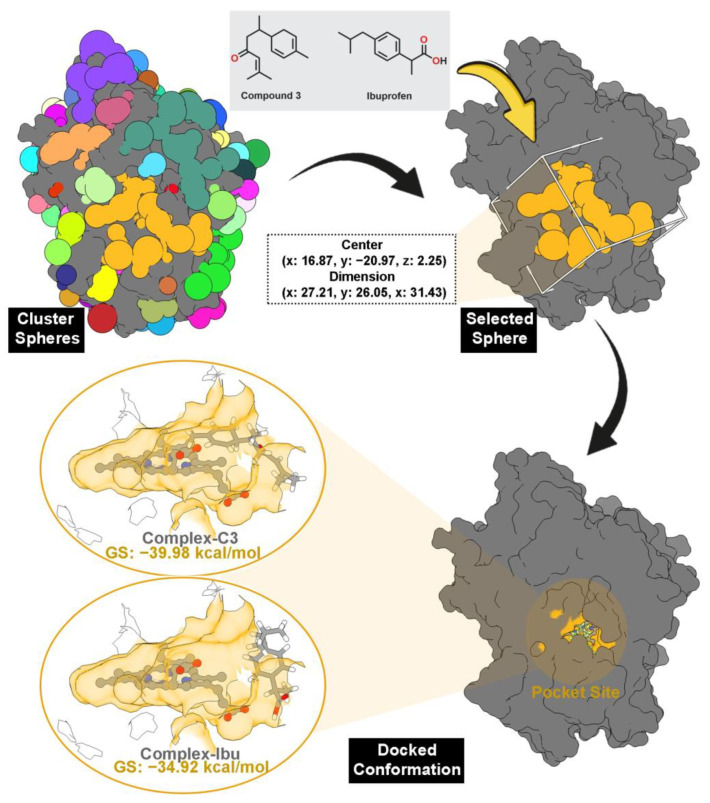
The pocket site was selected through cluster sphere selection (gold) from 108 cluster spheres. Note that each color represents each cluster sphere. The grid box was prepared by the coordinates of the selected cluster sphere. The docked conformation shows that each inhibitor binds well to the MPO pocket site. The MPO pocket site is shown in gold color.

Pocket site identification was carried out using sphere selection based on cluster analysis (Figure 3). A total of 108 cluster spheres were generated and ranked using the DOCK6 program [24]. Cluster 1, corresponding to the region with the highest probability of ligand binding, was selected as the initial coordinate for molecular docking. This cluster coincided precisely with the heme-binding cavity of MPO, consistent with its crystallographic active site.

Molecular docking results revealed that all the tested inhibitors fit well within the pocket, establishing stable interactions directly with the heme cofactor. The calculated grid scores (GS) correlated with the experimental anti-inflammatory data (Table 1), indicating strong agreement between in silico and in vitro findings. Compound **3** (C3) displayed the most favorable binding energy (−39.98 kcal/mol), surpassing the reference ibuprofen (−34.92 kcal/mol), suggesting stronger thermodynamic affinity for the MPO active site. The docked conformation of C3 (Figure 3) was subsequently used as the initial structure of MD simulations to further assess complex stability and binding behavior.

#### 3.4.2. Conformational Dynamics: Stability, Flexibility, and Rigidity

The docked conformations of each ligand–protein complex (compound **3** and ibuprofen) were subjected to MD simulations to evaluate their conformational stability and dynamic interactions with MPO. Additional simulations were performed for the Apo form of MPO (without heme or inhibitor) and the MPO–heme complex to provide comparative insight into intrinsic protein flexibility. The simulations aimed to capture the dynamic behavior of the inhibitors within the active site and assess the impact of ligand binding on protein stability. Structural parameters, including root-mean-square displacement (RMSD), root-mean-square fluctuation (RMSF), and radius of gyration (RoG), were analyzed over 100 ns trajectories using the *cpptraj* module of AmberTools25 [42].

**Figure 4:**
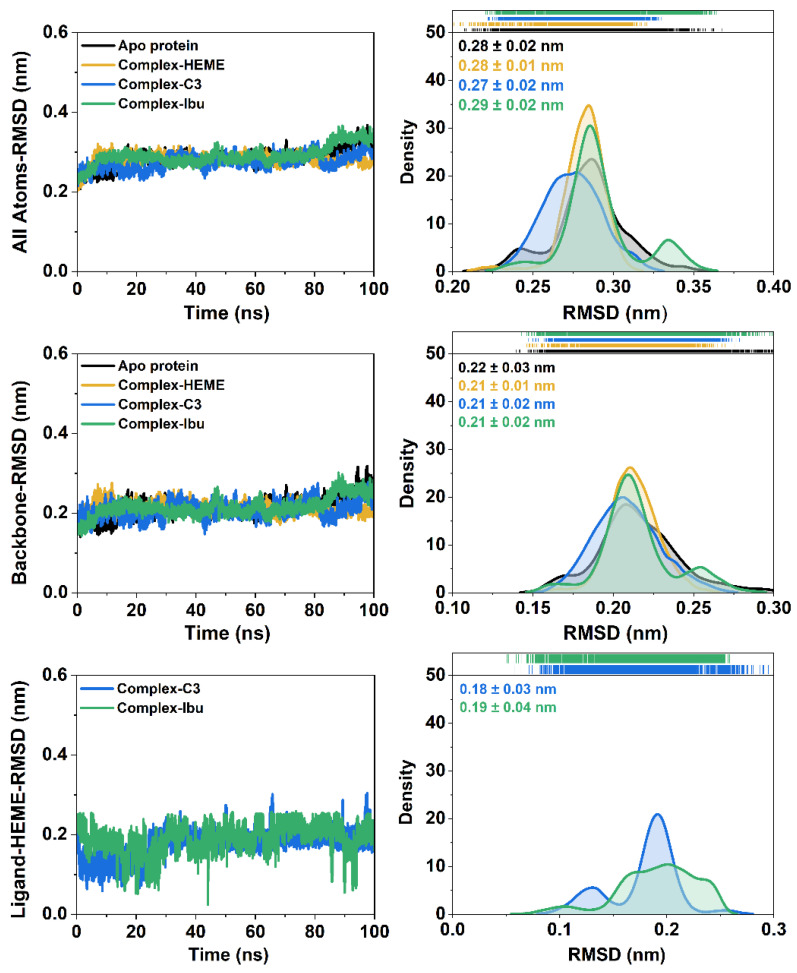
The root-mean-squared deviation (RMSD) of each system is plotted over 100 ns. The RMSD distribution is plotted using kernel smoothing. In particular, the RMSD of the backbone is calculated on the Cα, C, N, and O atoms of amino acid residues.

System stability during the 100 ns simulations was assessed through the RMSD analyses of all atoms, backbone atoms, and the heme ligand (Figure 4). All systems exhibited stable trajectories with RMSD fluctuations ≤ 0.4 nm, indicating well-equilibrated dynamics. Among the simulated systems, the C3-MPO complex showed the highest stability, with an all-atom RMSD distribution of ~0.20–0.34 nm and an average of 0.27 ± 0.02 nm, lower than those observed in the other complexes. These results suggested that binding of compound **3** enhances the overall stability of the ligand–heme–protein assembly.

**Figure 5:**
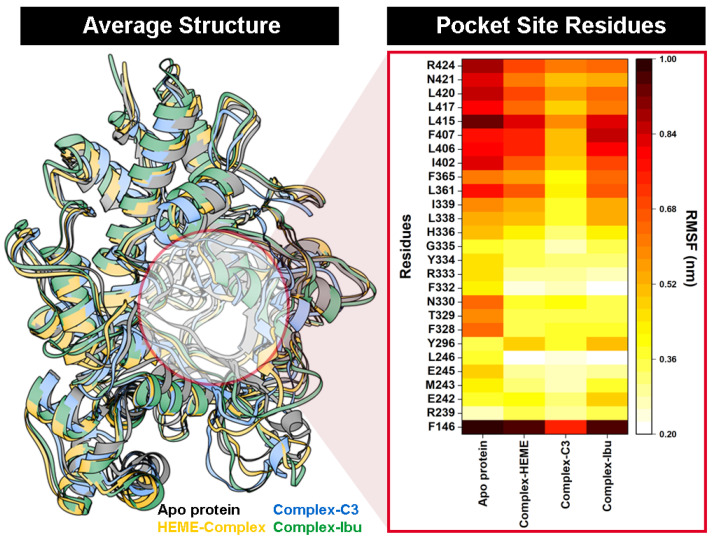
The *cpptraj* analysis using 100 ns of trajectories during the simulation: Average structure of each system. The heat-map distribution shows the flexibility of amino acid residues at the MPO pocket site.

RMSF analysis further supported these observations. Residues within the binding pocket displayed reduced flexibility upon interaction with compound **3**, as reflected by lower RMSF values in the heat-map distribution over the 100 ns trajectory (Figure 5). The restricted motion of key active-site residues implied stable and persistent interactions between compound **3** and MPO, consistent with the strong binding affinity observed in the docking results.

**Figure 6:**
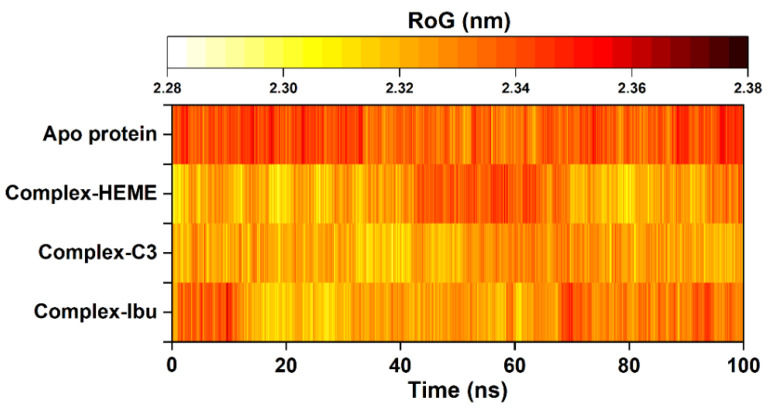
The radius of gyration (RoG) was plotted along 100 ns trajectories using the *cpptraj* tool. The RoG fluctuation is identified from the lowest (white) to the highest (black).

To further evaluate structural rigidity and compactness, the RoG was calculated for all simulated systems. RoG provides insights into the overall folding stability of the protein throughout the 100 ns trajectories [43]. The results revealed a clear correlation between RMSD and RoG, reflecting the stabilizing effect of inhibitor binding within the pocket site. Among all systems, the C3-MPO complex exhibited the lowest and most stable RoG values (2.33 ± 0.01 nm), followed by the MPO–heme complex (2.33 ± 0.01 nm), complex-Ibu (2.33 ± 0.01 nm), and the Apo protein (2.34 ± 0.01 nm) (Figure 6). The slightly reduced RoG observed for the C3-MPO complex suggested a more compact and well-folded structure, consistent with its lower RMSD and reduced residue fluctuations. These findings indicated that ligand binding, particularly by compound **3**, enhances the structural integrity and conformational stability of MPO during the simulation period.

#### 3.4.3. Binding Orientations: Energy Decomposition and Free Energy Binding

**Figure 7:**
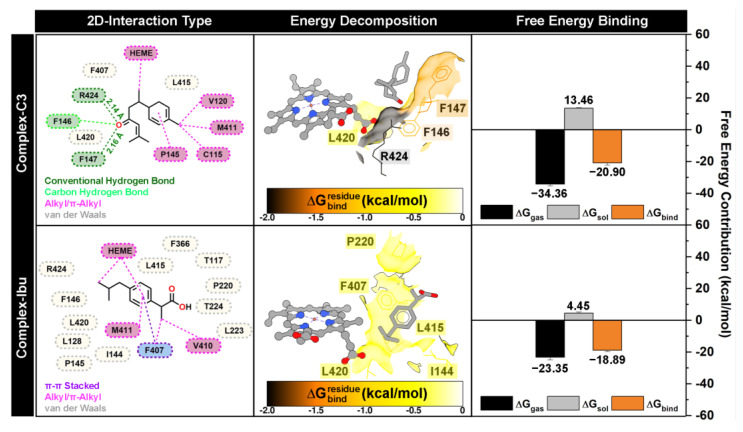
The complex conformation is extracted from the last 10 ns trajectories. The inhibitor-protein interaction is visualized by 2D-interaction types. The energy decomposition (${∆G}_{\mathrm{bind}}^{\mathrm{residue}}$) and free energy binding (∆G_bind_) were calculated using the MM-PBSA approach.

The stability of all complex systems was confirmed by the absence of significant RMSD fluctuations over the simulation period (Figure 3). Therefore, the last 10 ns of each trajectory were used for binding energy analyses, including residue-wise energy decomposition (${∆G}_{\mathrm{bind}}^{\mathrm{residue}}$) and total binding free energy (∆G_bind_) (Figure 7). Calculations were performed using the MM-PBSA approach implemented in the *MMPBSA.py* tool of AmberTools25 [28,44].

Binding orientation analysis revealed that both compound **3** and ibuprofen occupy positions near the heme and MPO pocket sites. The dominant interactions observed included conventional hydrogen bonds, carbon–hydrogen bonds, hydrophobic interactions (alkyl/π–π stacking), and van der Waals contacts. Interestingly, compound **3** formed two conventional hydrogen bonds with F147 and R424, acting as hydrogen bond acceptors, which likely play an important role in stabilizing the ligand–protein complex [45]. Residue-wise energy decomposition identified eight amino acids (I144, F146, F147, P220, F407, L415, L420, and R424) as significant contributors to ligand stabilization, defined by ${∆G}_{\mathrm{bind}}^{\mathrm{residue}}$ < −0.5 kcal/mol. Among these, F147 (−1.38 kcal/mol) and R424 (−1.97 kcal/mol) contributed most substantially, consistent with their involvement in hydrogen bonding with compound **3**. These findings provide a mechanistic basis for the inhibitory activity of compound **3**, suggesting that interactions with these residues are critical for suppressing ROS activity and exerting anti-inflammatory effects.

Furthermore, total binding free energy analysis indicated that compound **3** (ΔG_bind_ = −20.90 kcal/mol) binds more strongly than the control ibuprofen (ΔG_bind_ = −18.89 kcal/mol), reflecting favorable contributions from both gas-phase interactions (ΔG_gas_) and solvation effects (ΔG_sol_). Overall, the computational results, including docking scores, residue-wise energy decomposition (${∆G}_{\mathrm{bind}}^{\mathrm{residue}}$), and ΔG_bind_, correlated well with *in vitro* anti-inflammatory data (Table 1), confirming that compound **3** exhibits superior inhibitory potential compared to the reference compound.

## 4. Conclusions

In summary, this study provides a comprehensive evaluation of the phytochemical composition, anti-inflammatory activity, and molecular interactions of *Curcuma longa* fractions. GC-MS profiling revealed a chemically diverse set of sesquiterpenes and curcuminoids, with solvent polarity markedly influencing the yield and distribution of major constituents such as turmerones, zingiberene, and curlone. Bioassay-guided isolation from the dichloromethane (DCM) fraction yielded three key bioactive compounds, including bisacurone (**1**), didemethoxycurcumin (**2**), and β-turmerone (**3**), of which compound **3** exhibited the strongest anti-inflammatory activity compared to the standard drug ibuprofen. These biological findings were in line with molecular docking and dynamics simulations, which demonstrated stable and energetically favorable interactions between compound **3** and the MPO active site. The pronounced ROS inhibition observed for the DCM fraction, together with the potency of β-turmerone, highlights the importance of solvent polarity and phytochemical synergy in shaping the bioactivity. Additionally, the absence of acute toxicity in the brine shrimp assay indicates a favorable safety for the evaluated fractions. Collectively, these results underscore that *C. longa* exerts its anti-inflammatory effects through a coordinated network of curcuminoid and non-curcuminoid metabolites acting on multiple molecular targets. Looking ahead, emerging target-identification technologies could further deepen mechanistic insights. PROTAC-based target discovery probes enable proximity-induced capture of protein interactors in live-cell systems, facilitating unbiased identification of molecular targets for small phytochemicals. Complementary chemical proteomics platforms, including activity-based protein profiling (ABPP), thermal proteome profiling (TPP), and quantitative MS-based interaction mapping, provide powerful strategies to dissect the direct binding partners and downstream signaling pathways modulated by turmeric sesquiterpenes and curcuminoids [46,47]. Applying these tools in future studies would allow systematic elucidation of the molecular networks underlying the anti-inflammatory actions of *C. longa* bioactives identified herein, extending current insights beyond ROS inhibition and MOP binding.

## Figures and Tables

**Table 1:** Anti-inflammatory activity of turmeric fractions and isolated compounds.

Sample	IC_50_ (µg/mL) ± SD
*n*-Hexane fraction	5.4 ± 1.2
DCM fraction	≤0.4
EtOAc fraction	1.3 ± 0.3
*n*-Butanol fraction	8.7 ± 0.8
Aqueous fraction	NA ^b^
Didemethoxycurcumin **2**	45.5 ± 0.8
β-Turmerone **3**	4.7 ± 0.6
Ibuprofen ^a^	11.2 ± 1.9

^a^ Positive control; ^b^ NA: not active.

## Data Availability

The original contributions presented in this study are included in the article/Supplementary Materials. Further inquiries can be directed to the corresponding author.

## Supplementary Materials

The following supporting information can be downloaded at: https://www.mdpi.com/article/10.3390/foods14244205/s1. Figure S1. (+)-FAB-MS spectrum of bisacurone (**1**). Figure S2. ^1^H NMR spectrum of bisacurone (**1**) (CD_3_OD, 600 MHz). Figure S3. ^13^C NMR spectrum of bisacurone (**1**) (CD_3_OD, 150 MHz). Figure S4. LR-EI-MS spectrum of didemethoxycurcumin (**2**). Figure S5. ^1^H NMR spectrum of didemethoxycurcumin (**2**) (CD_3_OD, 500 MHz). Figure S6. ^13^C NMR spectrum of didemethoxycurcumin (**2**) (CD_3_OD, 125 MHz). Figure S7. (+)-FAB-MS spectrum of β-turmerone (**3**). Figure S8. ^1^H NMR spectrum of β-turmerone (**3**) (CD_3_OD, 500 MHz). Figure S9. ^13^C NMR spectrum of β-turmerone (**3**) (CD_3_OD, 125 MHz). Figure S10. GC-MS chromatogram of *n*-hexane fraction of *Curcuma longa* L. Figure S11. GC-MS chromatogram of dichloromethane fraction of *Curcuma longa* L. Table S1. Cytotoxicity activity (brine shrimp lethality assay). Table S2. Identified compounds from the *n*-hexane fraction of *Curcuma longa* L. using GC-MS. Table S3. Identified compounds from the dichloromethane fraction of *Curcuma longa* L. using GC-MS.
