# Supplementary material for: Anti-Inflammatory Activity-Guided Isolation and In Silico Validation of Turmeric (Curcuma longa L.) Phytochemicals
Source: Foods. 2025 Dec 7;14(24):4205. doi: 10.3390/foods14244205 (PMC12731395; doi:10.3390/foods14244205)
Supplement: Supplementary file 1 [file foods-14-04205-s001.zip › foods-3984070-supplementary.pdf]

SUPPLEMENTARY MATERIALS

Anti-inflammatory Activity-Guided Isolation and in Silico Validation of Turmeric (*Curcuma longa* L.) Phytochemicals

Zhuldyz Uvaniskanova [1,†], Salar Hafez-Ghoran [2,3,†,*], Muhammad Ikhlas Abdjan [4], Bel Youssouf G. Mountessou [5], Fatemeh Taktaz [2], Fadjar Mulya [6], Gulnaz A. Seitimova [1,*] and Muhammad Iqbal Choudhary [3,7]

1. Faculty of Chemistry and Chemical Technology, Al-Farabi Kazakh National University, Almaty 050040, Kazakhstan;
2. Laboratory for Functional Foods and Human Health, Center for Excellence in Post-Harvest Technologies, North Carolina Research Campus, North Carolina Agricultural and Technical State University, 500 Laureate Way, Kannapolis, NC 28081, USA;
3. H.E.J. Research Institute of Chemistry, International Center for Chemical and Biological Sciences (ICCBS), University of Karachi, Karachi 75270, Pakistan;
4. Department of Chemistry, Faculty of Mathematics and Natural Science, Universitas Negeri Surabaya, Surabaya 60213, Indonesia;
5. Department of Chemistry, Higher Teacher Training College, University of Yaoundé I, Yaoundé P.O. Box 47, Cameroon;
6. Nanotechnology Engineering, Faculty of Advanced Technology and Multidiscipline, Airlangga University, Surabaya 60115, Indonesia;
7. Dr. Panjwani Center for Molecular Medicine and Drug Research (PCMD), International Center for Chemical and Biological Sciences (ICCBS), University of Karachi, Karachi 75270, Pakistan.

†These authors contributed equally to this work.

*Correspondence:

Salar Hafez-Ghoran (S_Hafezghoran@yahoo.com & shafezghoran@ncat.edu),
Gulnaz A. Seitimova (gulnaz.seitimova@gmail.com & sitigulnaz@mail.ru).

Emails and ORCIDS

Zhuldyz Uvaniskanova (zhuldyz.uvaniskanova@gmail.com & 0000-0003-3719-4198),
Salar Hafez-Ghoran (S_Hafezghoran@yahoo.com & 0000-0001-5495-5556),
Muhammad Ikhlas Abdjan (muhammadabdjan@unesa.ac.id & 0000-0003-0783-5791),
Bel Youssouf G. Mountessou (mountessou@yahoo.com & 0000-0001-7220-3358),
Fatemeh Taktaz (ftaktaz@ncat.edu & 0000-0002-6521-4478),
Fadjar Mulya (fadjar.mulya@ftmm.unair.ac.id & 0000-0001-6539-3747),
Gulnaz A. Seitimova (gulnaz.seitimova@gmail.com & 0000-0002-5157-1255),
Muhammad Iqbal Choudhary (Iqbal.choudhary@iccs.edu & 0000-0001-5356-3585).

Abstract

Turmeric (*Curcuma longa* L., Zingiberaceae) is a widely consumed spice and functional food valued for its bioactive constituents. Using an activity-guided strategy, this study identified the dichloromethane fraction as the most potent anti-inflammatory fraction, exhibiting markedly stronger inhibition of reactive oxygen species (ROS) production than ibuprofen ($IC_{50} \leq 0.4$ *vs.* 11.2 μg/mL). Bioassay-guided purification yielded bisacurone (**1**), didemethoxycurcumin (**2**), and β-turmerone (**3**), with compounds **1** and **2** reported here for the first time in this fraction. Among them, β-turmerone displayed the strongest anti-inflammatory activity (IC_{50} = 4.7 μg/mL), consistent with *in silico* docking and molecular dynamics analyses revealing greater binding affinity and complex stability with myeloperoxidase (ΔG_{bind} = –20.90 *vs.* –18.89 kcal/mol for ibuprofen). Gas chromatography-mass spectrometry (GC-MS) profiling revealed a phytochemical profile dominated by turmerones and curlone, correlating with the observed bioactivity. None of the fractions exhibited acute toxicity in brine shrimp lethality assays, indicating a favorable preliminary safety profile. Our findings demonstrate the value of activity-guided isolation combined with computational validation for identifying turmeric-derived bioactives with promising nutraceutical potential, warranting further *in vivo* evaluation.

Keywords: *Curcuma longa* L.; β-Turmerone; Bioassay-guided isolation; Anti-inflammatory activity; Dynamic simulations.

**Table of content**	**Page**
**Figure S1.** (+)-FAB-MS spectrum of bisacurone (**1**).	3
**Figure S2.** ^{1}H NMR spectrum of bisacurone (**1**) (CD_3OD, 600 MHz).	4
**Figure S3.** ^{13}C NMR spectrum of bisacurone (**1**) (CD_3OD, 150 MHz).	5
**Figure S4.** LR-EI-MS spectrum of didemethoxycurcumin (**2**).	6
**Figure S5.** ^{1}H NMR spectrum of didemethoxycurcumin (**2**) (CD_3OD, 500 MHz).	6
**Figure S6.** ^{13}C NMR spectrum of didemethoxycurcumin (**2**) (CD_3OD, 125 MHz).	7
**Figure S7.** (+)-FAB-MS spectrum of β-turmerone (**3**).	8
**Figure S8.** ^{1}H NMR spectrum of β-turmerone (**3**) (CD_3OD, 500 MHz).	8
**Figure S9.** ^{13}C NMR spectrum of β-turmerone (**3**) (CD_3OD, 125 MHz).	9
**Figure S10.** GC-MS chromatogram of *n*-hexane fraction of *Curcuma longa* L.	9
**Figure S11.** GC-MS chromatogram of dichloromethane fraction of *Curcuma longa* L.	10
**Table S1.** Cytotoxicity activity (brine shrimp lethality assay).	10
**Table S2.** Identified compounds from the *n*-hexane fraction of *Curcuma longa* L. using GC-MS.	11
**Table S3.** Identified compounds from the dichloromethane fraction of *Curcuma longa* L. using GC-MS.	12

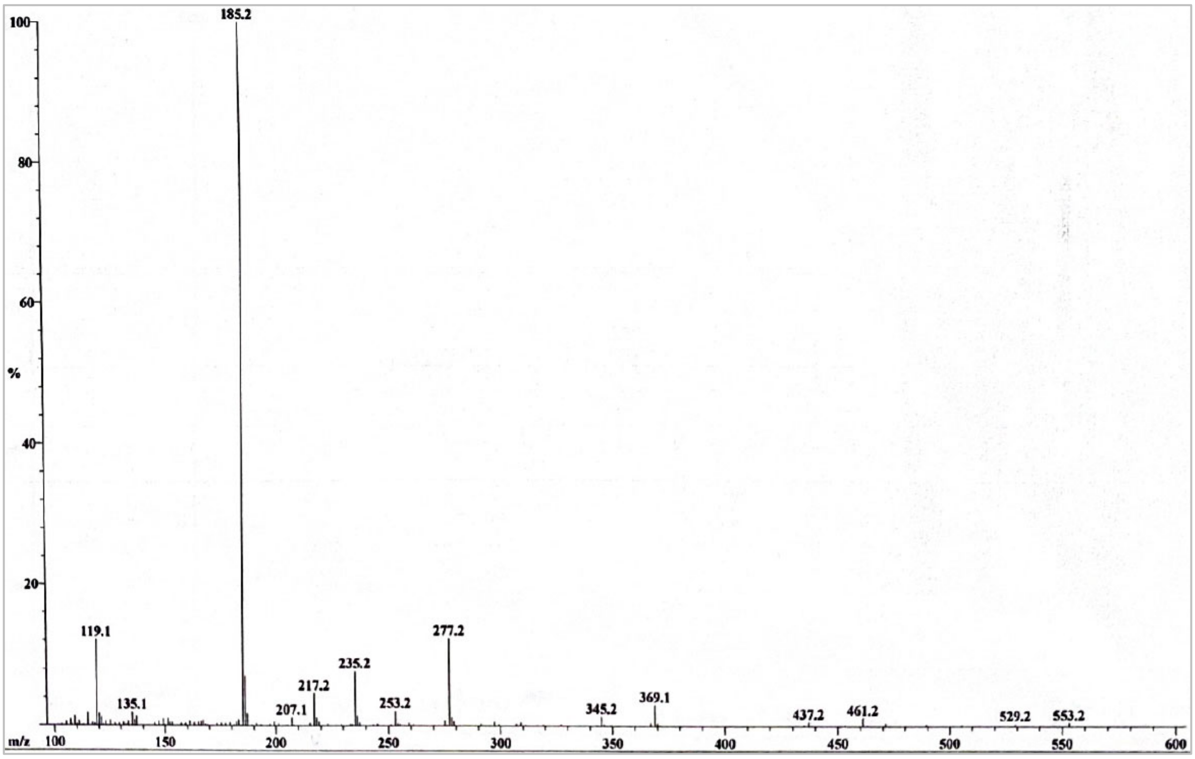

Figure S1. (+)-FAB-MS spectrum of bisacurone (**1**).

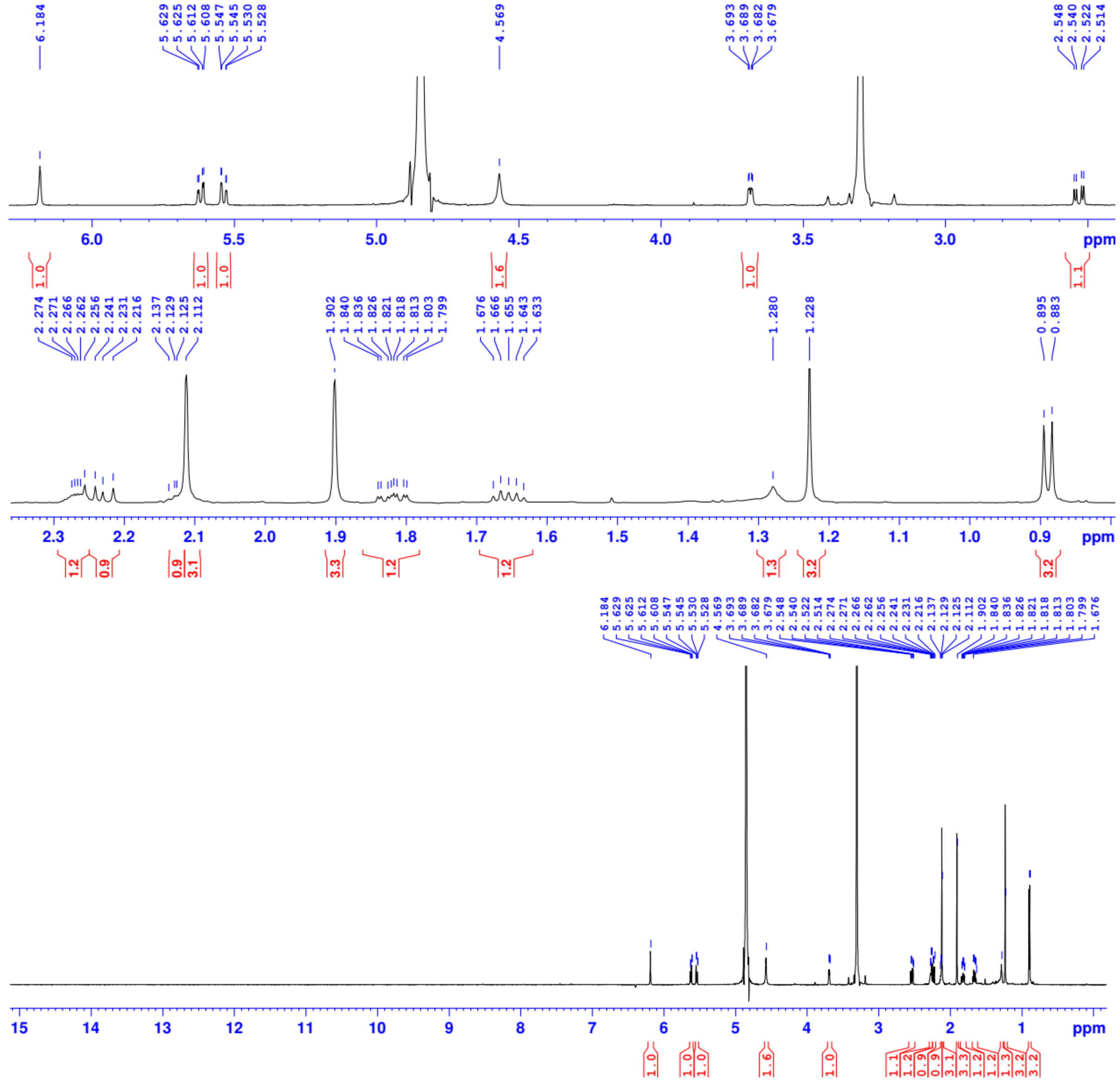

Figure S2. ^{1}H NMR spectrum of bisacurone (**1**) (CD_3OD, 600 MHz).

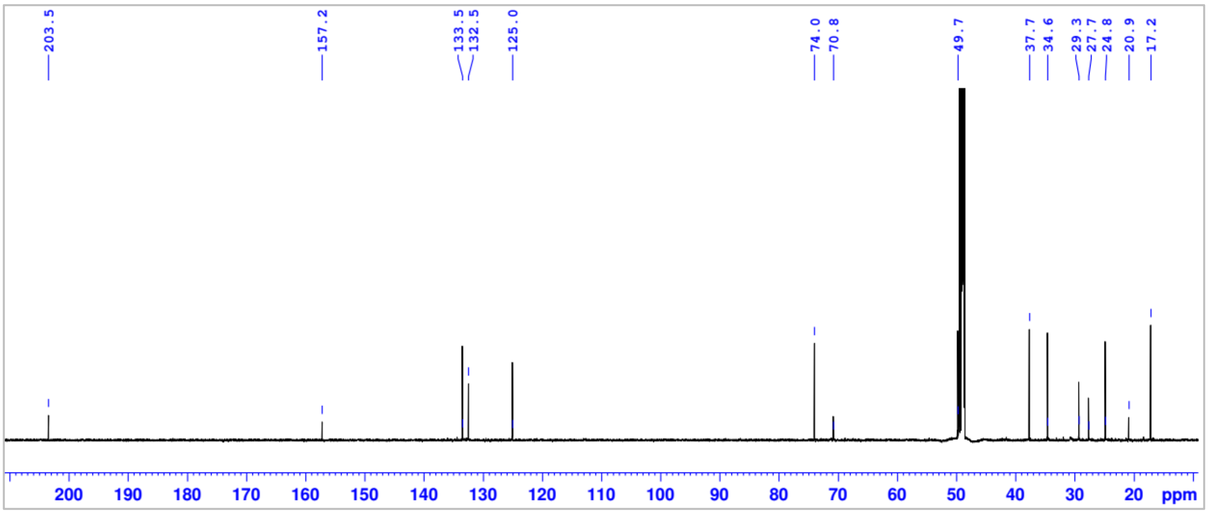

Figure S3. ^{13}C NMR spectrum of bisacurone (**1**) (CD_3OD, 150 MHz).

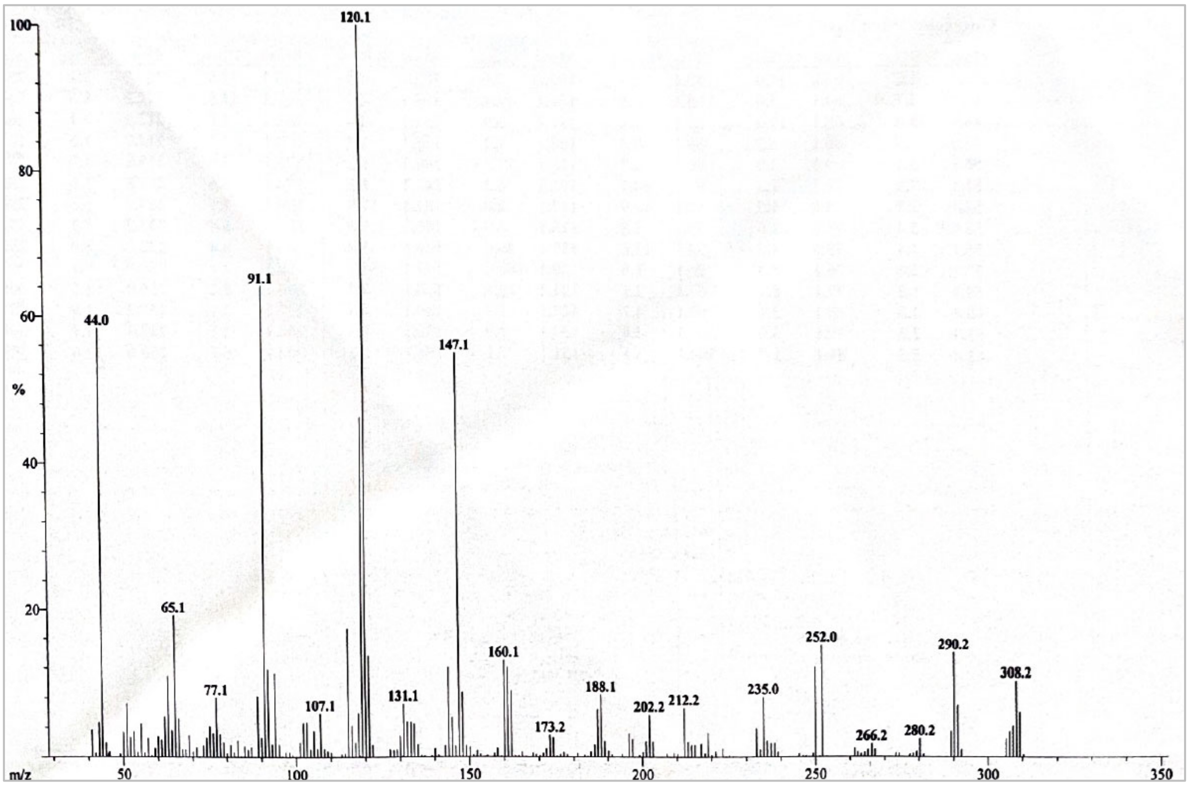

Figure S4. LR-EI-MS spectrum of didemethoxycurcumin (**2**).

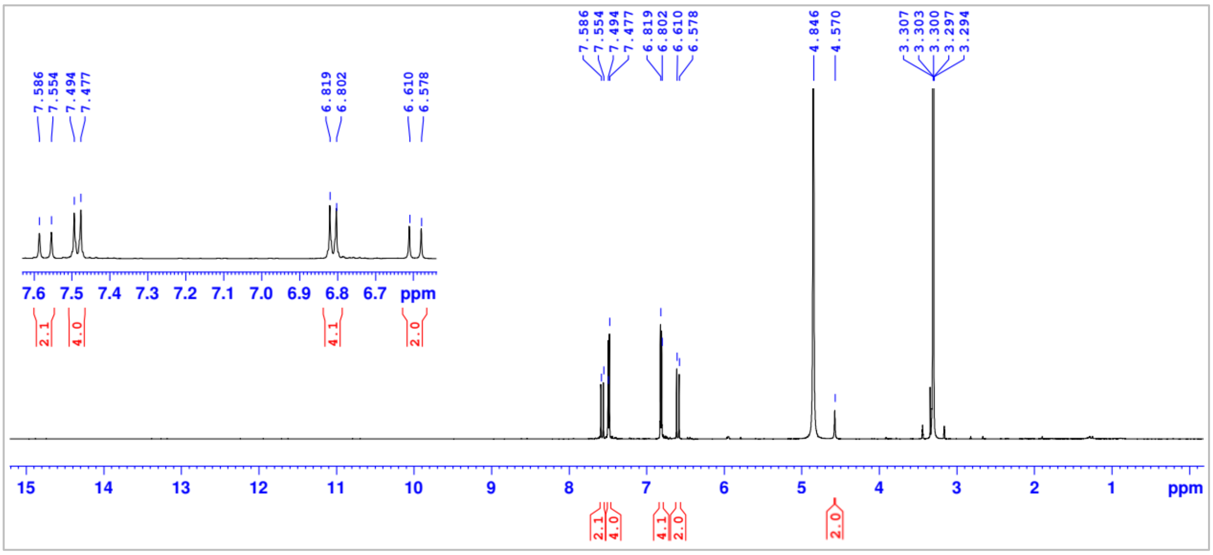

Figure S5. ^{1}H NMR spectrum of didemethoxycurcumin (**2**) (CD_3OD, 500 MHz).

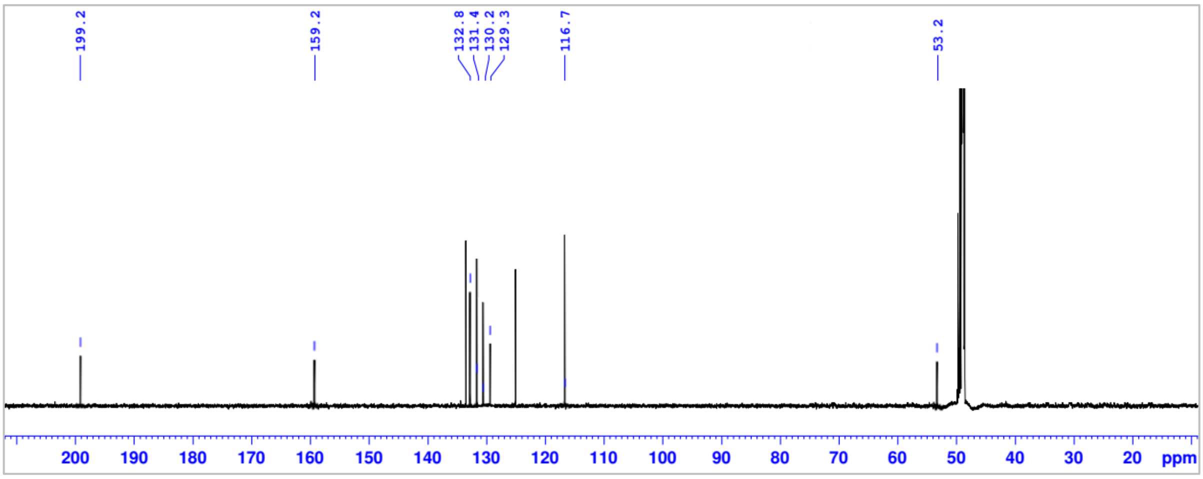

Figure S6. ^{13}C NMR spectrum of didemethoxycurcumin (**2**) (CD_3OD, 125 MHz).

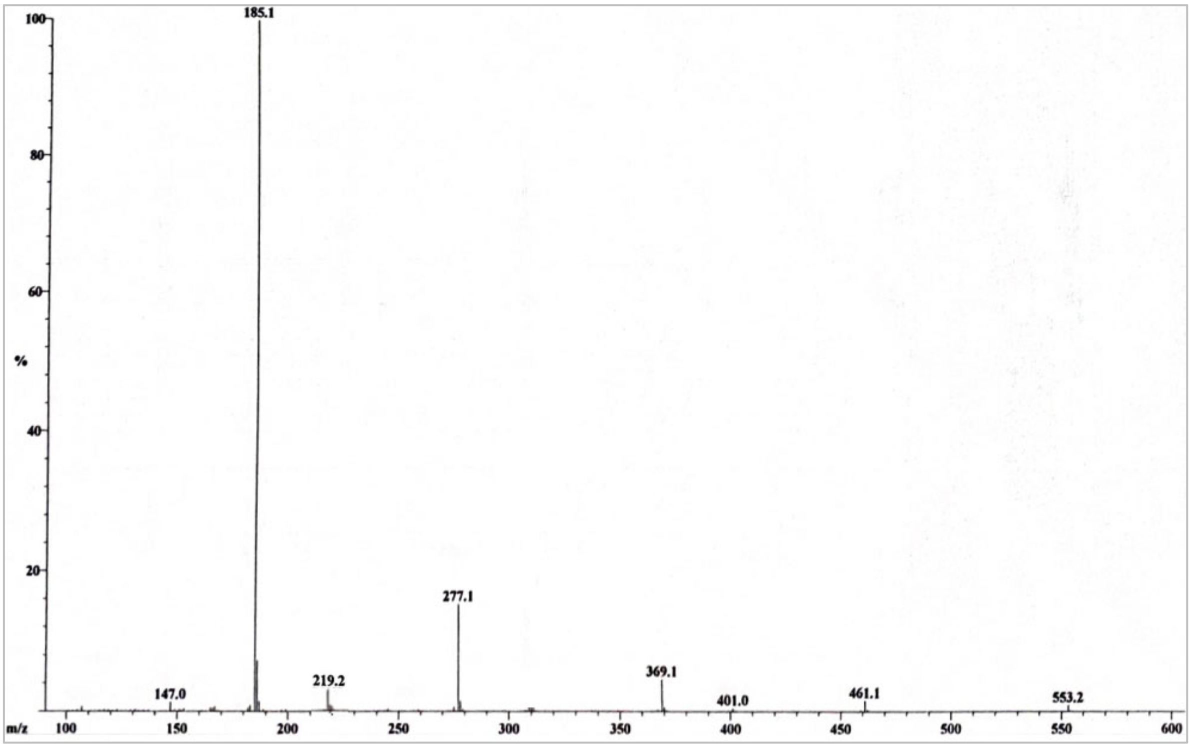

Figure S7. (+)-FAB-MS spectrum of β-turmerone (**3**).

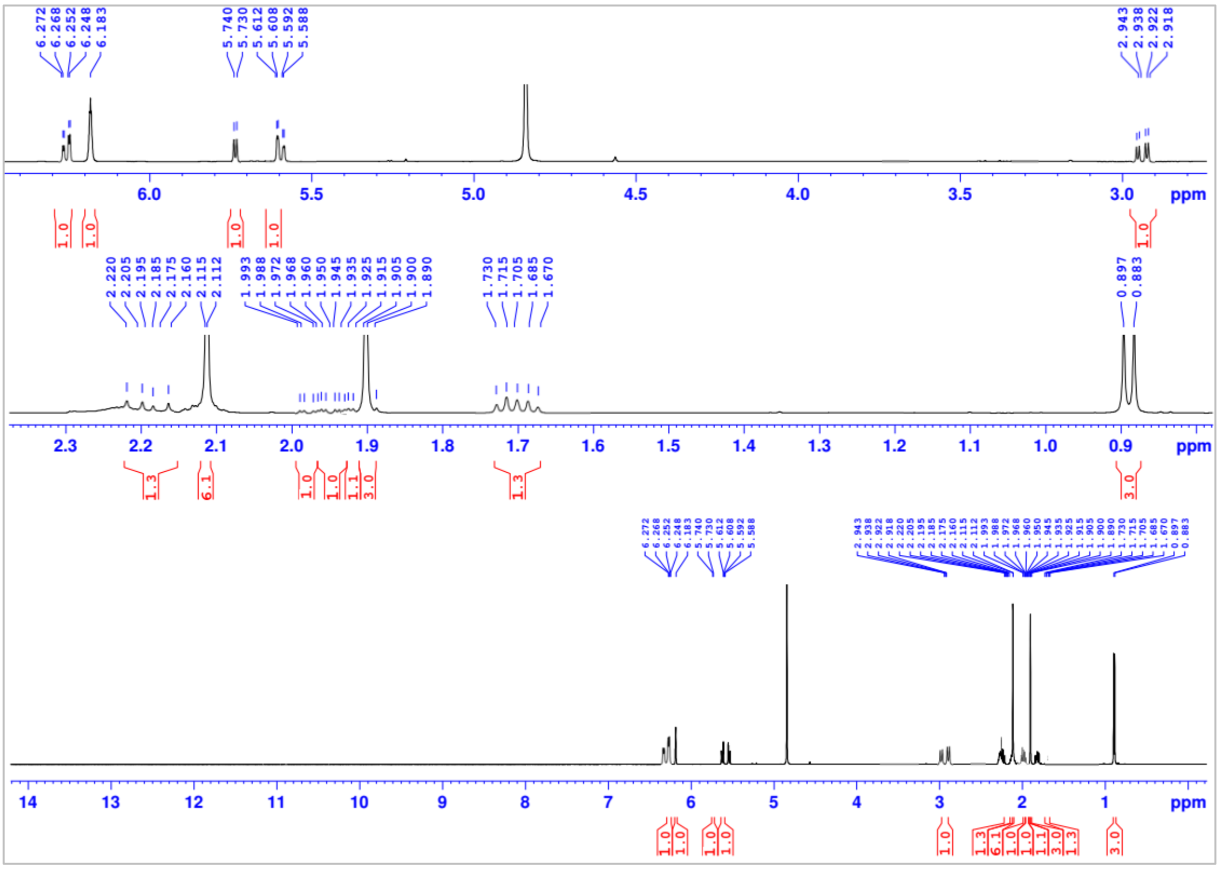

Figure S8. ^{1}H NMR spectrum of β-turmerone (**3**) (CD_3OD, 500 MHz).

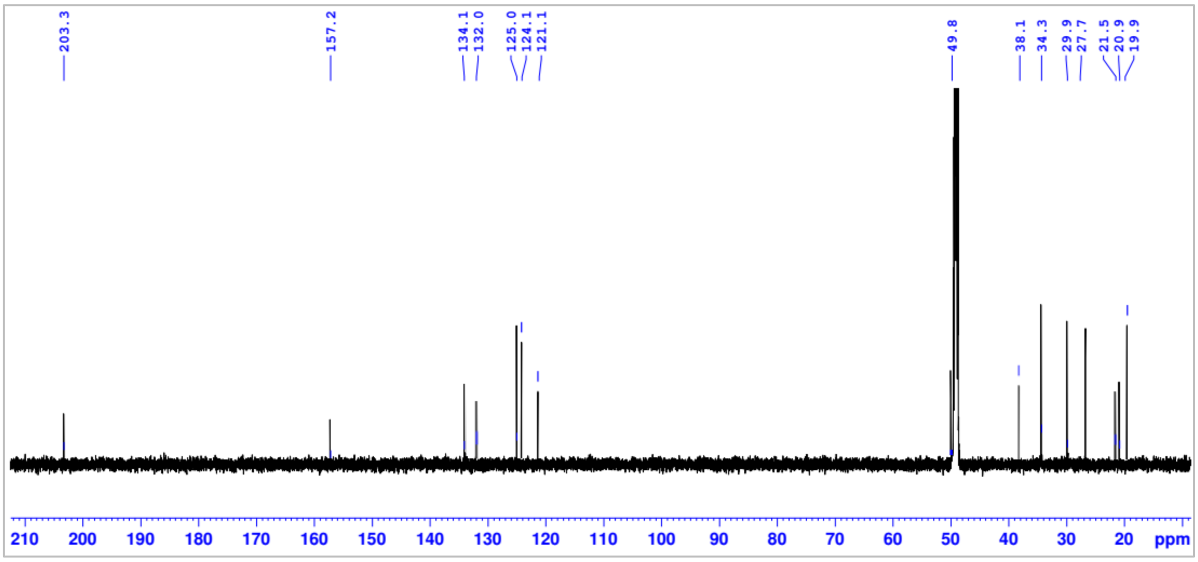

Figure S9. ^{13}C NMR spectrum of β-turmerone (**3**) (CD_3OD, 125 MHz).

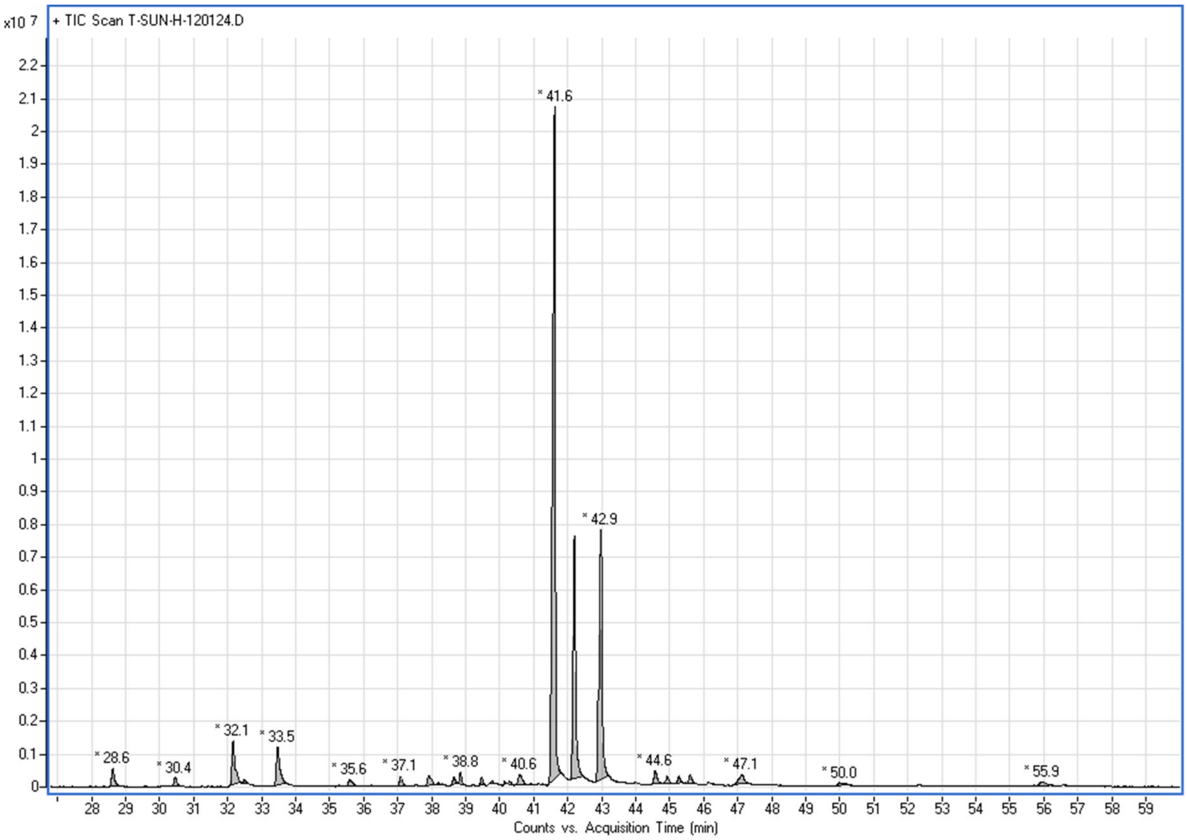

Figure S10. GC-MS chromatogram of *n*-hexane fraction of *Curcuma longa* L.

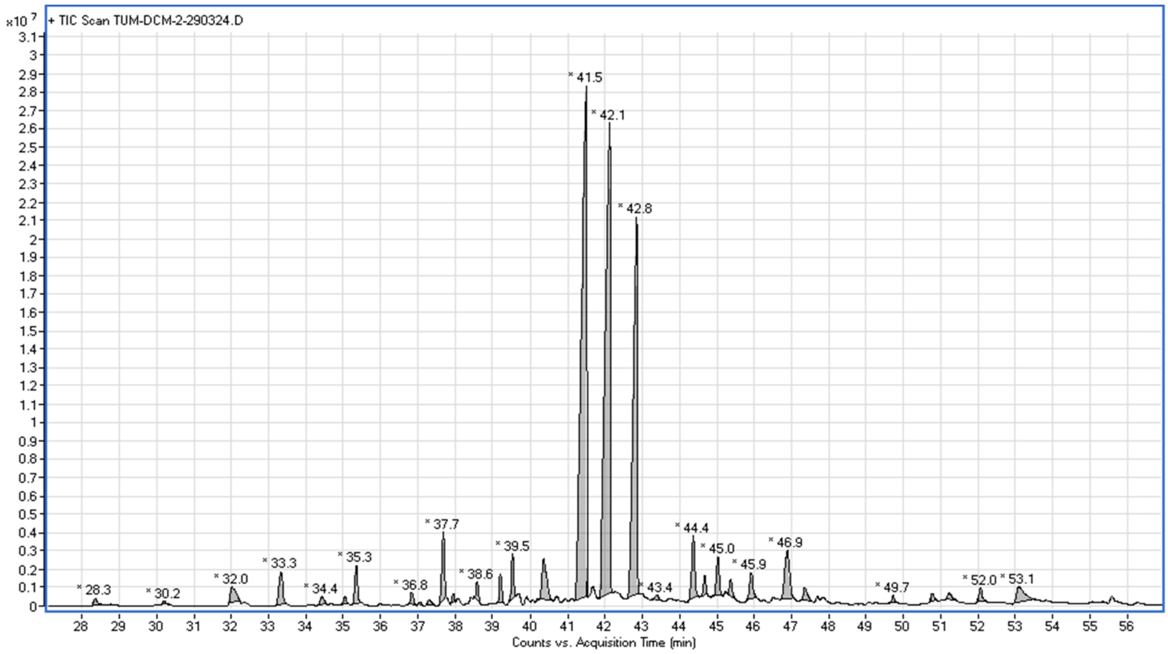

Figure S11. GC-MS chromatogram of dichloromethane fraction of *Curcuma longa* L.

Table S1. Cytotoxicity activity (brine shrimp lethality assay)

**Sample**	**Dose (µg/mL)**	**No. of Shrimps**	**No. of Survivors**	**Mortality, %**	**STD Drug**	**Mortality, %**
n-Hexane fr.	10	30	30	0		
	100	30	30	0		
	1000	30	30	0		
DCM fr.	10	30	30	0		
	100	30	30	0		
	1000	30	30	0		
EtOAc fr.	10	30	30	0		
	100	30	30	0	Etoposide	70
	1000	30	30	0		
n-Butanol fr.	10	30	30	0		
	100	30	30	0		
	1000	30	29	3.34		
Aqueous fr	10	30	30	0		
	100	30	30	0		
	1000	30	30	0		

Table S2. Identified compounds from the *n*-hexane fraction of *Curcuma longa* L. using GC-MS.

R_t (min)	%	Compound's name
28.6	1.26	Caryophyllene
30.4	0.64	*α*-Caryophyllene
32.1	3.5	Zingiberene
32.5	0.23	*β*-Bisabolene
33.5	3.4	*β*-Sesquiphellandrene
35.6	0.68	*trans*-Nerolidol
37.1	0.7	Epiglobulol
37.9	0.92	*α*-Bisabolol
38.2	0.19	*trans*-*α*-Bergamotol
38.6	0.46	Tumerone
38.8	0.71	*α*-Bisabolol
39.5	0.5	cis-*α*-Bergamotol
39.8	0.16	*E*-Nuciferol
39.9	0.07	cis,*α*-Santalol
40.1	0.15	Curlone
40.3	0.16	(7a-Isopropenyl-4,5-dimethyloctahydroinden-4-yl) methanol
40.6	1.02	*α*-Bergamotol
40.9	0.1	Bergamotol
41.6	46.74	Tumerone
42.2	16.02	*Ar*-tumerone
42.9	17.03	Curlone
44.6	0.99	1,8-dimethyl-4-(1-methylethyl)-spiro[4.5]dec-8-en-7-one
44.9	0.54	1-Methyl-6-(3-methylbuta-1,3-dienyl)-7-oxabicyclo[4.1.0]heptane
45.2	0.44	Ethyl citral
45.6	0.6	6-Isopropenyl-4,8a-dimethyl-4a,5,6,7,8,8a-hexahydro-1H-naphthalen-2-one
47.1	1.2	*α*-Santalol
50	0.47	Hexadecanoic acid ethyl ester
55.9	0.69	Linoleic acid ethyl ester
92	0.18	Ethyl *iso*-allocholate
97.4	0.25	Ethyl *iso*-allocholate

Table S3. Identified compounds from the dichloromethane fraction of *Curcuma longa* L. using GC-MS.

R_t (min)	%	Compound's name
28.3	0.32	Caryophyllene
30.2	0.14	*α*-Caryophyllene
32	1.13	Zingiberene
33.3	1.58	*β*-Sesquiphellandrene
34.4	0.41	Vanillin
35	0.28	Widdrol
35.3	1.19	(E)-3,7,11-trimethyl-1,6,10-Dodecatrien-3-ol
36.8	0.47	Thujopsene
37	0.08	Limonen-6-ol, pivalate
37.3	0.24	Caryophyllene oxide
37.7	2.07	Dihydrocurcumene
37.9	0.23	*trans*-*α*-Bergamotol
38.6	0.46	Widdrol
39.2	0.92	*trans*-*α*-Bergamotol
39.5	1.43	Tumerone
40.4	2.16	*trans*-*α*-Bergamotol
41.5	30.07	Tumerone
42.1	26.57	*Ar*-tumerone
42.8	16.47	Curlone
43.4	0.19	*E*-Nuciferol
44.4	2.25	(E)-1-(2,3-dimethyl-1,3-butadienyl)-2,2,6-trimethyl-7-oxabicyclo[4.1.0]heptane
44.7	0.56	Caryophyllene oxide
45	1.28	(2-nitro-2-propenyl)-Cyclohexane
45.4	0.55	6-Isopropenyl-4,8a-dimethyl-4a,5,6,7,8,8a-hexahydro-1H-naphthalen-2-one
45.9	1.15	5-methyl-2-(1-methyl-1-phenylethyl)-cyclohexanol
46.9	2.71	3,4-di[1-butenyl]-tetrahydrofuran-2-ol
47.3	0.7	7-(1,3-Dimethylbuta-1,3-dienyl)-1,6,6-trimethyl-3,8-dioxatricyclo[5.1.0.0(2,4)]octane
49.7	0.3	Ethyl palmitate
50.8	0.35	2,5-Octadecadiynoic acid methyl ester
51.2	0.33	4,4aα,5α,8α-tetrahydro-5,8-dimethyl-5,8-epoxy-3H-2-benzopyran
52	0.57	6Z-2,5,5,10-Tetramethyl-undeca-2,6,9-trien-8-one
53.1	1.59	Ethyl (2E)-3-(4-hydroxy-3-methoxyphenyl)-2-propenoate
91.6	0.45	Sitostenone
96.6	0.77	Cholest-4-ene-3,6-dione
